# COVID-19 Plasma Extracellular Vesicles Increase the Density of Lipid Rafts in Human Small Airway Epithelial Cells

**DOI:** 10.3390/ijms24021654

**Published:** 2023-01-14

**Authors:** Sara Darwish, Lauren P. Liu, Tanya O. Robinson, Spurthi Tarugu, Anna H. Owings, Sarah C. Glover, Abdel A. Alli

**Affiliations:** 1 Department of Physiology and Aging, University of Florida College of Medicine, Gainesville, FL 32610, USA; 2 Departments of Medicine and Cell and Molecular Biology, University of Mississippi Medical Center, Jackson, MS 39216, USA; 3 Department of Medicine, Division of Nephrology, Hypertension, and Renal Transplantation, University of Florida College of Medicine, Gainesville, FL 32610, USA

**Keywords:** COVID-19, extracellular vesicles, lipid rafts, small airway epithelial cells

## Abstract

Severe acute respiratory syndrome coronavirus 2 (SARS-CoV-2) virus is the causative agent of the COVID-19 disease. COVID-19 viral infection can affect many cell types, including epithelial cells of the lungs and airways. Extracellular vesicles (EVs) are released by virtually all cell types, and their packaged cargo allows for intercellular communication, cell differentiation, and signal transduction. Cargo from virus-infected cells may include virally derived metabolites, miRNAs, nucleic acids, and proteins. We hypothesized that COVID-19 plasma EVs can induce the formation of signaling platforms known as lipid rafts after uptake by normal human small airway epithelial cells (SAECs). Circulating EVs from patients with or without COVID-19 were characterized by nanoparticle tracking analysis, Western blotting using specific antibodies, and transmission electron microscopy. Primary cultures of normal human small airway epithelial cells were challenged with EVs from the two patient groups, and lipid raft formation was measured by fluorescence microscopy and assessed by sucrose density gradient analysis. Collectively, our data suggest that circulating EVs from COVID-19-infected patients can induce the formation of lipid rafts in normal human small airway epithelial cells. These results suggest the need for future studies aimed at investigating whether the increased density of lipid rafts in these cells promotes viral entry and alteration of specific signaling pathways in the recipient cells.

## 1. Introduction

Severe acute respiratory syndrome coronavirus 2 (SARS-CoV-2), also referred to as COVID-19, is an infectious disease that emerged in early 2020 and has rapidly spread across the globe, triggering a global health crisis and causing a multitude of perturbations [1]. Currently, the investigation of the virus and its long-term effects on lung health continues to be a priority due to its associated comorbidities, resulting in high mortality rates [2]. The primary infection initiation site is the epithelial mucosa of the upper respiratory tract and lungs, with a clinical presentation consisting of fever, cough, fatigue, and dyspnea [3,4]. SARS-CoV-2 is a 100 nm single-stranded RNA-enveloped virus. Its entry into host cells depends on the binding of the viral (S) spike protein to the angiotensin-converting enzyme 2 (ACE2) receptor, which is mainly expressed in alveolar epithelial cells. Later, activation of serine protease TMPRSS2 leads to a cascade of reactions, triggering infection [4,5]. Recent studies have shown that the infectivity of SARS-CoV-2 is heavily dependent on endocytic mechanisms and has been shown to undergo rapid endocytosis in cells expressing abundant levels of ACE2 [5].

Extracellular vesicles (EVs) include exosomes, microvesicles, and apoptotic bodies [6]. These vesicles are generated either from the budding of the plasma membrane or within the endosomal system through the fusion of multivesicular endocytic compartments [7]. EVs have the potential to be used as a therapeutic tool, given their ability to participate in immune modulation and act as delivery vehicles containing various molecules [8]. EVs are present in conditioned media from cultured cells [9] and in different types of biological fluids, including serum, plasma, urine, and saliva [10]. Urinary EVs are mainly released from epithelial cells within the nephron and are found abundantly in urine [11]. Plasma and serum EVs are released from many different cell types and are found in the systemic circulation [12]. A subtype of intraluminal vesicles (ILVs) known as exosomes are lipid-bound nano-sized organelles derived from a fusion of multivesicular bodies (MVBs) and have been thought to function primarily as a means of excreting cellular waste material, although later studies have suggested their possible role in biochemical signaling [13]. All types of EVs are rich in various types of proteins, metabolites, nucleic acids, and lipids [14,15]. Additionally, EVs have been shown to be enriched in bioactive lipids, signaling lipids, and lipids associated with lipid rafts [16,17]. In conjunction with their ability to mediate the transfer of membrane components such as proteins, existing studies have effectively shown their role in the transmission of viral infectious agents [18]. 

Whereas the potential role of EVs in SARS-CoV-2 has been explored in other studies, a deeper understanding of the functional role of EVs and their potential contribution to the viral transmission of SARS-CoV-2 requires additional investigation. The primary objective of our study is to elucidate the active role of COVID-19-derived EVs in the trajectory of the disease by investigating their potential contribution to lipid raft formation in human small epithelial airway cells. Lipid rafts are structures located within the plasma membrane that are composed of sphingolipids, cholesterol, and proteins. Their function includes the regulation of membrane signaling and trafficking [19,20], indicating that they might contribute to the viral transmission of SARS-CoV-2. Our findings presented here highlight how COVID-19 plasma EVs can increase the density of lipid rafts in recipient human small airway epithelial cells (SAECs) to potentially promote viral entry in these cells.

## 2. Results

### 2.1. Patient Characteristics

The two study groups included *n* = 6 patients with COVID-19 and *n* = 6 patients without COVID-19. Background data on each patient in the two groups are presented in Table 1, including age, gender, race, BMI, and smoking history. 

### 2.2. Serum BUN, Creatinine, and Electrolyte Levels in Each COVID-19 and Non-COVID-19 Patient

**Figure 1:**
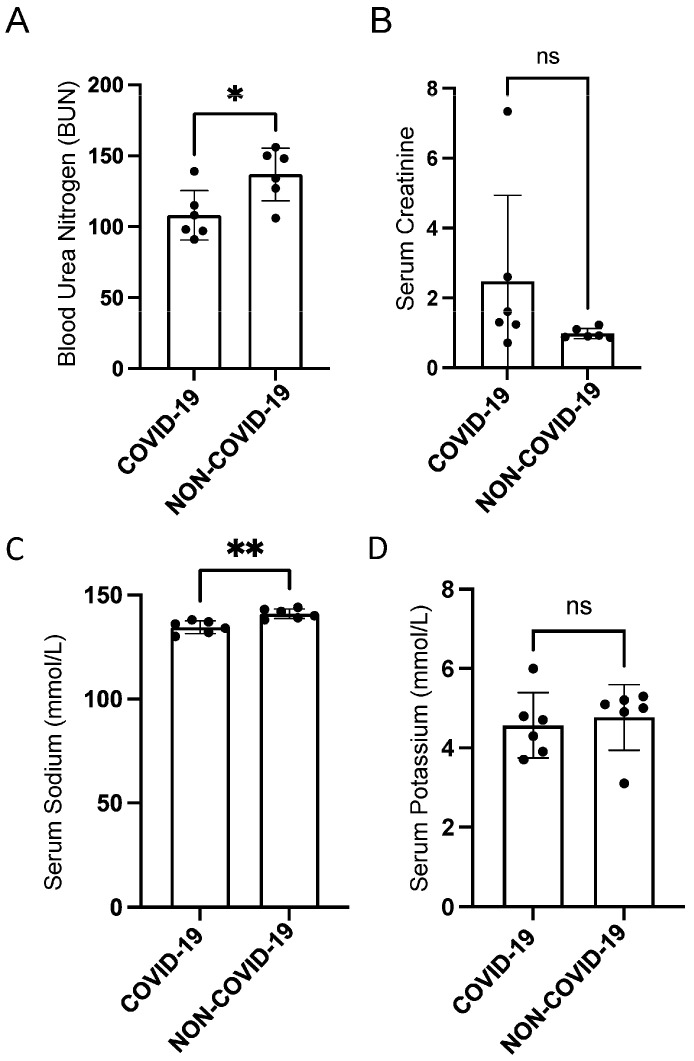
Comparison of serum BUN, creatinine, and electrolytes in COVID-19 and non-COVID-19 patients. (**A**) Blood urea nitrogen (BUN), (**B**) serum creatinine, (**C**) serum sodium, and (**D**) serum potassium levels in patients with and without COVID-19. *n* = 6 COVID-19 patients and *n* = 6 non-COVID-19 patients, respectively. An unpaired *t*-test was performed. * *p*-value < 0.05; ** *p*-value < 0.01. ns refers to a non-significant difference between the groups.

As shown in Figure 1, our cohort of COVID-19 patients had lower levels of BUN (Figure 1A) and serum sodium (Figure 1C) compared to the non-COVID-19 group. There was no significant difference in serum creatinine or serum potassium between the two groups, as shown in Figure 1B,D.

### 2.3. Characterization of EVs Isolated from the Plasma of COVID-19 and Non-COVID-19 Patients

**Figure 2:**
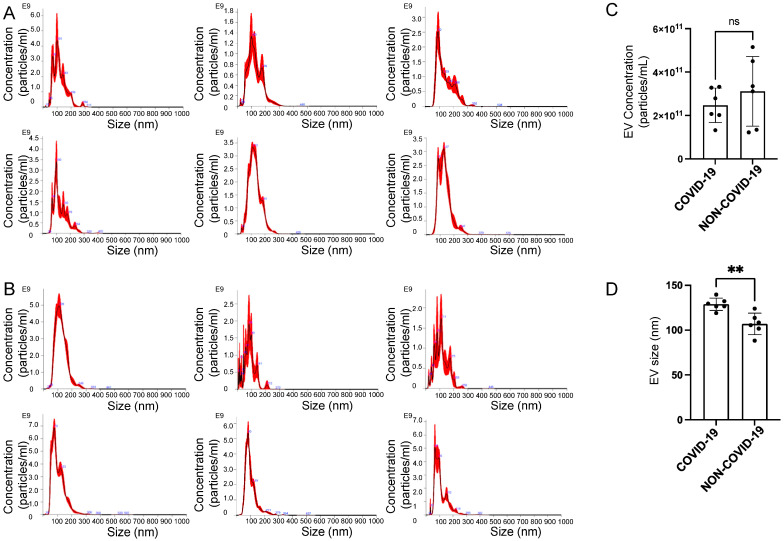
Nanoparticle tracking analysis of EVs isolated from the plasma of patients with and without COVID-19. (**A**) Mean peak profiles for EVs isolated from the plasma of COVID-19 patients. (**B**) Mean peak profiles for EVs isolated from the plasma of non-COVID-19 patients. (**C**) Summary plot of EV concentrations from the COVID-19 and non-COVID-19 groups. (**D**) Summary plot of EV size from the COVID-19 and non-COVID-19 groups. An unpaired *t*-test was performed. ** *p*-value < 0.01. ns refers to a non-significant difference between the groups.

The concentration and size of each plasma EV sample from the COVID-19 and non-COVID-19 groups were measured by nanoparticle tracking analysis. The average peak profile of each EV sample from both groups showed that concentrations were in the range of 1.22–5.15 × 10^11^ particles/mL, and the majority of the EV particles were less than 200 nm in diameter (Figure 2A, B). Although there was not a significant difference in concentration between the COVID-19 and non-COVID-19 groups (Figure 2C), the EVs isolated from the plasma of COVID-19 patients were larger than those isolated from non-COVID-19 patients (Figure 2D).

**Figure 3:**
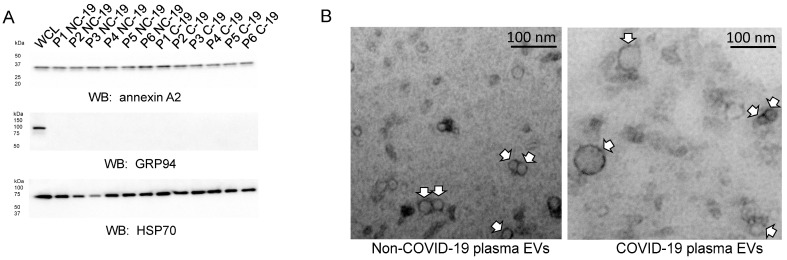
Western blot analysis of plasma EVs (pEVs) from patients with and without COVID-19. (**A**) Western blots for annexin A2, glucose-regulated protein 94 (GRP94) (endoplasmic reticulum marker and control for the absence of lysed cells and aggregated protein), and HSP70. WCL, whole cell lysate from normal human fibroblasts (BJ cells). (**B**) Representative transmission electron microscopy images of plasma EVs from COVID-19 and non-COVID-19 patients. White arrows indicate individual EVs. Images are representative of *n* = 6 different batches of non-COVID-19 plasma EVs (pNC-19) and *n* = 6 different batches of COVID-19 plasma EVs (pC-19).

The EVs from each of the two groups were then further characterized for the enrichment of known EV markers by Western blot analysis. Each EV preparation from the COVID-19 and non-COVID-19 group was found to be enriched in annexin A2 and HSP70 but not in the endoplasmic reticulum marker protein GRP94 (Figure 3A). Transmission electron microscopy was performed to corroborate the nanoparticle tracking results, showing that the EVs from the COVID-19 group were larger than those of the non-COVID-19 group, whereas the size of most of the EVs was less than 200 nm in diameter (Figure 3B). 

### 2.4. pEVs from COVID-19 Patients Increase Lipid Raft Density in Recipient SAECs 

**Figure 4:**
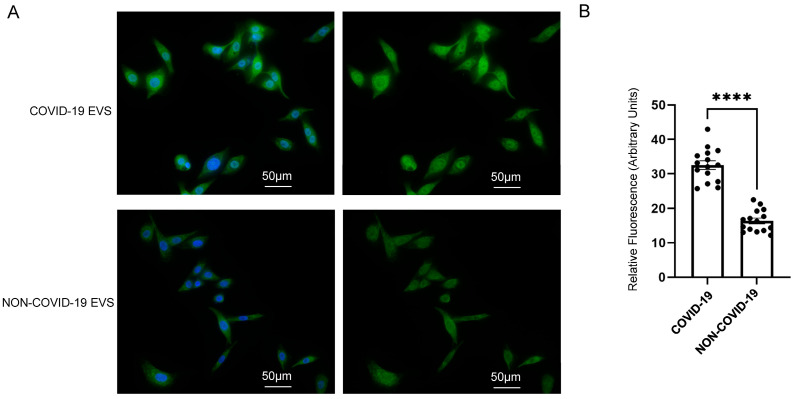
Immunofluorescence and analysis of lipid raft density after challenging human small airway epithelial cells (SAECs) with pEVs from patients with and without COVID-19. (**A**) Representative immunofluorescence microscopy of human SAECs challenged with pEVs from COVID-19 patients (top) and non-COVID-19 patients (bottom). Human SAECs were fixed, and lipid rafts were labeled with cholera toxin B subunit conjugated to FITC. DAPI was added to visualize nuclei. The cells were imaged at 40× using a wide-field Nikon microscope. The scale bar represents 50 μm, and each image was adjusted using the same post-processing settings (130 min and 1100 max) for the green channel. (**B**) Summary plot of the fluorescence intensity quantification in panel A. A total of 15 cells in each group were quantified using ImageJ 1.52a software. Similar results were observed in *n* = 3 independent experiments performed using 3 different batches of either COVID-19 plasma EVs or non-COVID-19 plasma EVs. An unpaired *t*-test was performed. **** *p*-value < 0.0001.

To investigate whether circulating EVs from COVID-19 patients can regulate lipid raft formation and signal transduction in normal human SAECs, we measured the density of lipid rafts in normal human SAECs challenged with EVs isolated from the plasma of COVID-19 patients compared to non-COVID-19 patients. Fluorescence microscopy and analysis showed that the density of lipid rafts was augmented in cells challenged by EVs from the COVID-19 group compared to the non-COVID-19 control group (Figure 4).

### 2.5. pEVs from COVID-19 Patients Increase MAL Enrichment with Lipid Rafts in Human SAECs

**Figure 5:**
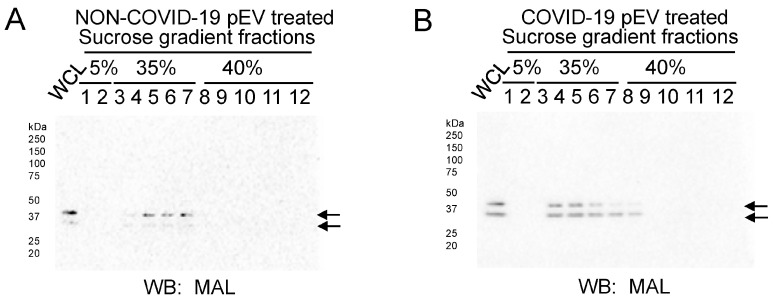
Sucrose density gradient analysis after challenging normal human small airway epithelial cells (SAECs) with pEVs from patients with and without COVID-19. (**A**) Western blot for MAL protein in twelve sucrose density gradient fractions after human SAECs were challenged with COVID-19 plasma EVs. (**B**) Western blot for MAL protein in twelve sucrose density gradient fractions after human SAECs were challenged with non-COVID-19 plasma EVs. WCL refers to whole cell lysate from SAECs as a positive control. Similar results were observed in *n* = 3 independent experiments performed using 3 different batches of non-COVID-19 plasma EVs and 3 different batches of COVID-19 plasma EVs.

MAL protein expression was augmented in lipid-raft-associated sucrose density gradient fractions after treating human SAECs with pEVs from COVID-19 patients compared to non-COVID-19 patients (Figure 5). 

### 2.6. COVID-19 pEVs Decrease the Permeability of FITC-Dextran in hAoEC and SAEC

**Figure 6:**
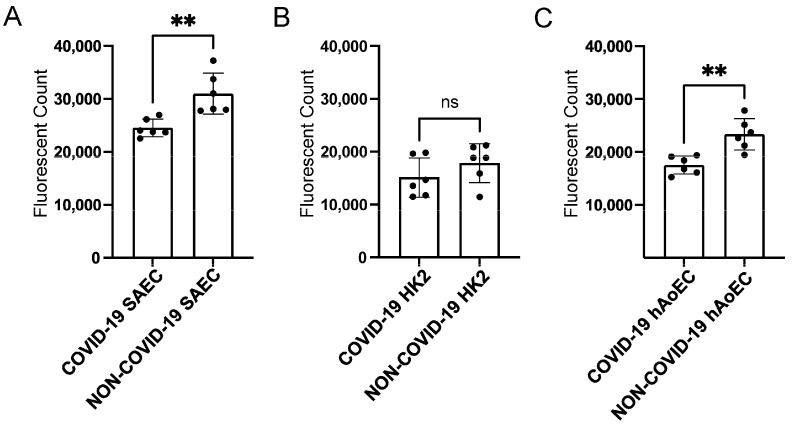
Permeability assay after challenging various human cell types with COVID-19 and non-COVID-19 pEVs. (**A**) Permeability of FITC-dextran in human SAECs challenged with pEVs from patients with or without COVID-19. (**B**) Permeability of FITC-dextran in human proximal tubule (HK2) cells challenged with pEVs from patients with or without COVID-19. (**C**) Permeability of FITC-dextran in human aortic endothelial cells (hAoECs) challenged with pEVs from patients with or without COVID-19. An unpaired *t*-test was performed. ** *p*-value < 0.01. ns refers to a non-significant difference between the groups.

To investigate whether pEVs alter the permeability of multiple human cell types, we performed a fluorescence permeability assay. Normal human small airway epithelial cells (SAECs), human proximal tubule cells (HK2s), and human aortic endothelial cells (hAoECs) were challenged with pEVs from COVID-19 and non-COVID-19 patients. As shown in Figure 6, pEVs from COVID-19 patients decreased the permeability of FITC-dextran in SAECs (Figure 6A) and hAoECs (Figure 6C).

## 3. Discussion

**Figure 7:**
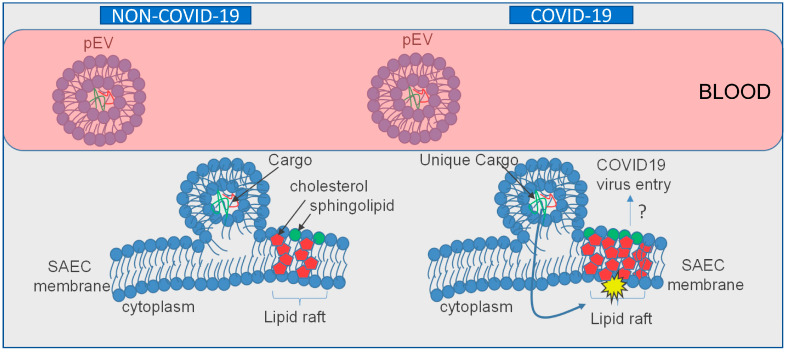
Proposed model for the role of EVs isolated from the plasma of non-COVID-19 patients compared to those from the plasma of COVID-19 patients in SAECs. The cargo enriched in pEVs of COVID-19 patients contain a unique overall cargo, which, upon uptake by recipient small airway epithelial cells, primes lipid rafts for the activation of various intracellular signaling pathways. Presumably, the stimulation of lipid rafts or the resulting activation of intracellular signaling pathways promotes COVID-19 viral entry into the cells. A “?” represents a possible pEV dependent mechanism for COVID-19 virus entry.

Here, we present evidence from multiple approaches demonstrating that circulating EVs from COVID-19 patients are capable of increasing the density of lipid rafts in recipient human small airway epithelial cells (SAECs) relative to pEVs from non-COVID-19 patients. This increase in lipid raft density in SAECs challenged with COVID-19 pEVs is likely to result in the activation of various signaling pathways (Figure 7). We first showed, by fluorescence microscopy, that there is a greater density of cholera-toxin-subunit-B-labeled lipid rafts in human SAECs challenged with pEVs isolated from the plasma of COVID-19 patients compared to those from non-COVID-19 patients. Secondly, we showed that the density of the lipid raft protein MAL (and potentially MAL2) is augmented in light-density sucrose gradient fractions from human SAECs challenged with pEVs from COVID-19 patients compared to those from non-COVID-19 patients. Although we used a MAL antibody, the results suggest that the doublet at ~37 kDa seen in our Western blots corresponds to heavy glycosylated forms of MAL and, presumably, MAL2. MAL2 was previously shown by other groups to migrate at a similar molecular weight [21,22]. 

Typically, there are numerous comorbidities and factors including patient demographics, age, BMI, sex, behaviors, and medication that can influence the packaging of cargo within EVs and/or their release and subsequent effects in recipient cells. Our COVID-19 patient cohort and non-COVID-19 patient cohort included both male and female adults in the age range of 45 to 80 who either had a history of smoking or never smoked at all. The patients from both groups were on various medications, which may have influenced the release of EVs from different cell types and, presumably, the packaged molecules within the EVs. For example, drugs that induce hypoxic stress [23], reduce blood pressure and edema [24], induce vasodilation [25], stabilize mast cells [26], treat cancer [27], and lower blood glucose [28] have been shown to modulate the release of EVs from various cell types. It may be possible for various drugs to alter the EV enrichment of bioactive lipids such as sphingomyelins and phosphatidylethanolamines, which are associated with lipid rafts. Additional studies using SARS-CoV-2 pseudovirus to infect specific cell types may be helpful to further investigate and confirm the role of COVID-19 pEVs in modulating the organization of lipid rafts. 

Our data suggest that the augmentation of lipid rafts located within human SAECs triggered by COVID-19 plasma extracellular vesicles signifies their potential role in the viral transmission of SARS-CoV-2 and activation of other potential subsequent biochemical pathways. Lipid rafts have previously served as points of entry for viruses such as influenza and HIV [29], suggesting the possibility of a similar effect in SARS-CoV-2. As demonstrated, a notable difference in size between pEVs derived from our COVID-19 patients compared to non-COVID-19 patients was determined via transmission electron microscopy. Additionally, our electrolyte data are consistent with published studies. Similar to the results shown in a study by Lippi et al. [30], we also found lower sodium, potassium, and calcium levels in patients with severe COVID-19 disease. 

Importantly, published studies have shown that cholesterol-rich lipid rafts serve as platforms to concentrate various receptors, allowing for their interaction with the viral spike protein and subsequently promoting viral entry. Lu et al. showed that the angiotensin-converting enzyme-2 (ACE-2) is localized to lipid rafts and demonstrated the association with the ectodomain of SARS-CoV-2 S protein and lipid rafts in Vero E6 cells [31]. They also showed that the disruption of lipid rafts inhibits the infectivity of pseudotyped SARS-CoV-2 in these cells. Płóciennikowska et al. showed that human toll-like receptors (TLRs) are localized to lipid rafts [32], whereas Choudhury and Mukherjee showed that various forms of TLRs are involved in the docking of the S protein [33]. In another study, Wei et al. showed that the HDL scavenger receptor B type 1 promotes SARS-CoV-2 entry [34], whereas the depletion of cholesterol from cell membranes was shown by Radenkovic et al. to inhibit SAR-CoV-2 infection [35]. 

One limitation of our study is the small sample size, which restricts our ability to potentially investigate different variants of COVID-19. An additional limiting factor to consider is the constrained range of demographics. Further research with a larger sample size and a wider spectrum of demographics would be beneficial to further support the findings presented in this study. 

Taken together, the data presented here provide preliminary evidence for the potential role of circulating EVs in COVID-19 viral infection. Descriptive studies aimed at identifying differentially enriched molecules, including bioactive lipids, metabolites, and proteins, in EVs isolated from the plasma of COVID-19 and non-COVID-19 patients may offer valuable insight into the mechanism by which these EVs can promote lipid raft formation in recipient cells and alter signal transduction pathways and/or promote viral entry. 

## 4. Materials and Methods

### 4.1. Cell Culture

Human small airway epithelial cells (SAECs) (CC-2547) were purchased from Lonza (Walkersville, MD, USA) and cultured in SAEC medium (Lonza). Human proximal tubule (HK-2) cells were purchased from the ATCC (Manassas, VA, USA) and cultured in keratinocyte medium (Invitrogen (Gibco). Human aortic endothelial cells (hAoECs) were purchased from Cell Biologics (Chicago, IL, USA) and cultured in complete growth medium (Cell Biologics). Normal human BJ fibroblasts were a kind gift from Dr. J. W. Shay (University of Texas Southwestern Medical Center). All cell types were maintained at 37 °C in a 5% CO_2_ humidified incubator, and the medium was replaced every 3 days. SAECs, HK-2 cells, and hAoECs with a passage number of 2–5 were used for experiments. 

### 4.2. Participant Recruitment and Respiratory Sampling

Full participant characteristics are provided in a previously published study [36]. The UMMC Institutional Review Board approved the study under IRB#2020-0065. All participants or their legally authorized representative provided written informed consent. Briefly, participants were eligible for inclusion in the COVID-19 group if they were at least 18 years old; had a positive nasopharyngeal swab for SARS-CoV-2 by PCR; had COVID-19-related symptoms, including fever, chills, cough, shortness of breath, and sore throat; and weighed more than 110 lb. Participants were eligible for inclusion in the healthy group if they were at least 18 years old, had a current negative SARS-CoV-2 test (PCR or rapid antigen test), and weighed more than 110 lb. COVID-19 participants were classified according to the 8-level ordinal scale proposed by the WHO representing their peak clinical severity and level of respiratory support required. Blood samples were collected by a trained healthcare in dark-green-top tubes containing sodium heparin provider and processed within 1 h of collection. To collect plasma, tubes were centrifuged at 1200× *g* in a clinical-grade centrifuge for 10 min at room temperature. Plasma aliquots were stored at −20 degrees Celsius until used. 

### 4.3. Isolation of Extracellular Vesicles from Human Plasma Samples

EVs were isolated from plasma samples from patients with or without COVID-19. SmartSEC EV isolation kits (SSEC200A-1) (System Biosciences, Palo Alto, CA) were used according to the manufactures instructions with the following modifications. The plasma samples were subject to centrifugation at 3000× *g* for 10 min and then 400 μL of the supernatant was added to the top of the resin bed of a SmartSEC column after washing the beads. Three columns were used for each patient sample and the fractions were pooled together. 

### 4.4. Nanoparticle Tracking Analysis

The sizes and concentrations of extracellular vesicles were measured by a NanoSight NS300 machine (Malvern Panalytical, Malvern, UK) equipped with NTA 3.4 Build 3.4.4 Software (Malvern Panalytical) at 25 degrees Celsius. The samples were diluted with ultrapure 1× PBS (Thermo Fisher Scientific) at a ratio of 1:1000 before being fed into the NS300 machine using an automated infusion pump at a rate of 65.

### 4.5. SDS PAGE and Western Blotting

EVs were lysed in a 1:1 dilution with RIPA buffer (Thermo Fisher Scientific) before determining total protein concentration using a bicinchoninic acid protein assay (Thermo Fisher Scientific). Twenty micrograms of total protein was loaded into 4–20% Tris HCl polyacrylamide gels and resolved using the Criterion electrophoresis system (BioRad, Hercules, CA, USA). The resolved proteins were then transferred onto nitrocellulose membranes (GE Healthcare, Piscataway, NJ, USA) using the Criterion transfer system (BioRad). The membranes were blocked in a mixture of 5% non-fat milk and 1× TBS (TBS; BioRad) at room temperature for an hour on an automated rocker. The nitrocellulose membranes were washed twice with 1× TBS and incubated with primary antibodies (annexin A2 (8235; Cell Signaling), GRP94 (20292; Cell Signaling Technology), and HSP70 (ab228421; Abcam)) overnight on an automated rocker at 4 degrees Celsius. Next, the membranes were washed three times with 1× TBS and incubated with horseradish peroxidase-conjugated goat anti-rabbit secondary antibody (BioRad) at a 1:3000 dilution prepared in blocking solution for an hour on an automated rocker. The nitrocellulose membranes were washed four times with 1× TBS, incubated with ECL reagent (BioRad) for seven minutes, and developed on an imager (BioRad).

### 4.6. Transmission Electron Microscopy

A total of 25 μL of a 1:10 dilution of each sample (COVID-19 or non-COVID-19 EVs) was added to an equal volume of 4% paraformaldehyde in 1× PBS (Thermo Fisher Scientific). A 20 μL drop of the EV–paraformaldehyde solution was aliquoted onto a section of Parafilm; then, a Formvar carbon-coated grid (Ladd Research Industries; Williston, VT, USA) (membrane side down) was floated on top of the drop, and the sample was allowed to absorb for 20 min at room temperature. Next, the grid was transferred to a 50 μL drop of 1× PBS placed on a section of Parafilm, transferred to a 50 μL drop of 1% glutaraldehyde (Ladd Research Industries; Williston, VT, USA) in 1× PBS on another section of Parafilm, and allowed to incubate for 5 min at room temperature. The grid was then subjected to a series of 8 washes, each in a 50 μL drop of distilled water on a section of Parafilm for 2 min intervals. The grid was then incubated in a 50 μL drop of filtered 4% aqueous uranyl acetate (Ladd Research Industries) and 0.15 M oxalic acid (Sigma-Aldrich, St. Louis, MI, USA) solution (pH 7), placed on a different section of the Parafilm, and incubated for 5 min at room temperature. Afterwards, the grid was incubated in a 50 μL drop of a solution comprising one part filtered 4% aqueous uranyl acetate and nine parts methyl cellulose (Sigma-Aldrich) on a different section of Parafilm that was placed in a glass Petri dish on a cold plate and incubated for 10 min. Next, the grids were removed using a stainless-steel loop, blotted around the edges with Whatman no. 1 filter paper, and allowed to air dry for 5 min at room temperature. Finally, the grid was imaged using a Hitachi H-7600 transmission electron microscope (Hitachi High Technologies America, Inc., Clarksburg, MD, USA) with AMT imaging software (Advanced Microscopy Techniques Corporation).

### 4.7. SAEC Treatment and Fluorescence Microscopy

SAECs were plated on glass-bottom dishes (Mattek; Ashland, MA, USA) and cultured at 37 °C for 24 h. The cells were made quiescent by serum depravation in basal medium for 8 h before being challenged with 1–5 × 10^7^ particles/mL of EVs for 4 h. The cells were washed twice in 1× PBS and fixed in ice-cold methanol/acetone solution (1:1 ratio *v*/*v*) at −20 °C for 10 min. After a series of 3 washes with 1× PBS, cholera toxin B subunit (Sigma) was added to label lipid rafts, and DAPI (Vector Laboratories) was added to label nuclei. The cells were then imaged using a 40× objective on an inverted Nikon Ti-E widefield microscope. 

### 4.8. Sucrose Density Gradient Ultracentrifugation

Lipid rafts were isolated from cell lysates of SAECs by sucrose density gradient ultracentrifugation, as previously described [37]. Protein expression of lipid-raft- and non-lipid-raft-associated proteins was assessed by Western blotting. 

### 4.9. In Vitro Permeability Assay

A fluorescence-based in vitro permeability assay (ECM644) (Millipore, Burlington, MA, USA) was used to assess changes in permeability of FITC-dextran in multiple cell types. Human SAECs, human proximal tubule (HK2) cells, and human aortic endothelial cells (hAoECs) were plated on inserts and treated with 1–5 × 10^7^ particles/mL pEVs from COVID-19 or non-COVID-19 patients for 4 h before being treated with FITC-dextran. The plate was read at 485 nm (excitation) and 535 nm (emission) on a Tecan fluorescence plate reader (Switzerland) equipped with Magellan software.

### 4.10. Statistical Analysis

ImageJ Version 1.52 A was used to quantify immunofluorescence intensity. GraphPad Prism Version 9.4.1 was used to analyze the data and create the plots. An unpaired t-test was used to make comparisons between the COVID-19 group and the non-COVID-19 group. 

## 5. Conclusions

In conclusion, the data presented here suggest a new role for circulating EVs in the transmission of SARS-CoV-2. Descriptive studies aimed at identifying differentially enriched molecules, including bioactive lipids, metabolites, and proteins, in EVs isolated from the plasma of COVID-19 and non-COVID-19 patients may offer important insight into the mechanism by which these EVs can promote lipid raft formation in recipient cells and alter signal transduction pathways and/or promote viral entry. Future experiments should investigate the pathways involved in the induction of lipid rafts by the uptake of EVs in recipient cells and how the resulting changes in permeability modify SARS-CoV-2 infection in lung epithelial cells. Future studies should also explore the possible therapeutic potential of targeting specific molecules in pathways involved in the induction of lipid rafts by EVs from patients with COVID-19. 

## Figures and Tables

**Table 1:** Demographics of patients with or without COVID-19.

Patient Sample	COVID Status	Age	Gender	Race	BMI	Smoker
NON-COVID-19 P1	negative	51	M	Caucasian	42.95	Never
NON-COVID-19 P2	negative	49	F	Black	45.5	Never
NON-COVID-19 P3	negative	59	M	Black	24.6	Current
NON-COVID-19 P4	negative	54	F	Caucasian	51.4	Never
NON-COVID-19 P5	negative	45	F	Black	27.8	Prior
NON-COVID-19 P6	negative	59	F	Black	37.4	Never
COVID-19 P1	positive	52	F	Black	49.69	Never
COVID-19 P2	positive	79	M	Caucasian	17.5	Prior
COVID-19 P3	positive	71	M	Choctaw	39.38	Prior
COVID-19 P4	positive	65	M	Black	27.98	Prior
COVID-19 P5	positive	65	M	Black	29.5	Never
COVID-19 P6	positive	65	F	Black	44.92	Never

## Data Availability

The individual data points are presented in each plot within the figures.
